# From EGFR PTM network to TKI resistance: spatial subtypes and targeting in lung cancer

**DOI:** 10.20517/cdr.2025.231

**Published:** 2026-03-06

**Authors:** Birou Lai, Chang Xu, Siyi Lai, Mo Zhou, Hesheng Kong, Eryan Kong, Badrul Hisham Yahaya

**Affiliations:** ^1^The First Affiliated Hospital of Henan Medical University, Xinxiang 453100, Henan, China.; ^2^Department of Biomedical Sciences, Pusat Kanser Tun Abdullah Ahmad Badawi (PKTAAB), Universiti Sains Malaysia, Kepala Batas 13200, Penang, Malaysia.; ^3^Xinxiang Key Laboratory of Protein Palmitoylation and Major Human Diseases, Institute of psychiatry and neuroscience, Henan Medical University, Xinxiang 453003, Henan, China.

**Keywords:** Lung cancer, EGFR, TKI resistance, spatial PTM, subcellular trafficking, degradation

## Abstract

Lung cancer represents the most prevalent and lethal malignancy worldwide. Although tyrosine kinase inhibitors targeting the epidermal growth factor receptor (EGFR) demonstrate clinical efficacy, the emergence of resistance remains a major therapeutic obstacle. This review comprehensively examines how six key post-translational modifications (PTMs) of EGFR - phosphorylation, palmitoylation, ubiquitination, glycosylation, acetylation, and S-nitrosylation - collectively govern its signaling dynamics, protein turnover, and subcellular trafficking. Based on this mechanistic framework, we propose a novel classification of resistance subtypes: membrane-retained, degradation-evading, nuclear-localized, and mitochondrial-localized EGFR, each defined by distinct PTM signatures and spatial localization. Furthermore, we analyze the intricate crosstalk among these PTMs, revealing hierarchical and often cooperative relationships that ultimately determine the fate and function of EGFR. Our analysis suggests that targeting specific spatial PTM hubs or their interactive networks, rather than EGFR alone, offers a promising strategy to overcome resistance. We also emphasize the need to integrate multi-PTM profiling with spatial proteomics to inform precision combination therapies. This work proposes a shift in the therapeutic paradigm from mere kinase inhibition toward reprogramming the pathological PTM network underlying resistant lung cancer.

## INTRODUCTION

Lung cancer (LC) remains a global health burden, accounting for approximately 2 million new cases and 1.76 million deaths annually, making it the most common and deadliest malignancy worldwide^[1]^. However, most cases are detected only after the disease has progressed to an advanced or metastatic stage, resulting in poor survival outcomes^[2]^. Therefore, the identification of novel therapeutic targets is urgently needed.

The main cause of LC is smoking, accounting for about 85% of all cases worldwide^[3]^. Smoking introduces a multitude of carcinogens that directly and/or indirectly induce DNA damage within cells. This damage disrupts cellular homeostasis, impairs the regulation of key processes such as cell growth, motility, metastatic potential, apoptosis, and DNA repair, thereby elevating the risk of LC^[4,5]^. LC is commonly associated with genetic mutations driving tumor growth and progression^[6]^, of which the epidermal growth factor receptor (EGFR) mutations are the second most common oncogenic driver alteration in LC^[7]^.

EGFR is a transmembrane receptor tyrosine kinase that plays a critical role in regulating cell growth, proliferation, and differentiation^[8,9]^. EGFR tyrosine kinase inhibitors (TKIs) serve as a first-line treatment for patients with non-small cell lung cancer (NSCLC) harboring sensitizing EGFR mutations^[10]^. However, despite an initial favorable response in most patients, resistance inevitably develops, ultimately limiting the long-term efficacy of these therapies^[11]^.

Several post-translational modifications (PTMs) critically modulate EGFR activity, localization, and stability, serving both as a major mechanism underlying TKI resistance and as a potential therapeutic avenue to overcome it^[10]^. Recent studies indicate that compared with TKI-sensitive cells, third-generation TKI-resistant cells exhibit substantial alterations at both the proteome and phosphoproteome levels^[12]^. Furthermore, dysregulated ubiquitination could facilitate the evasion of EGFR from degradation during EGFR-TKI resistance, thereby sustaining EGFR overexpression^[13]^. For example, proteolysis-targeting chimeras (PROTACs) can induce the degradation of EGFR through the ubiquitin-proteasome pathway^[14,15]^. Additionally, the Melanoma Cell Adhesion Molecule (MCAM), a highly glycosylated type I transmembrane protein, has also been found to be up-regulated in EGFR-TKI resistant lung adenocarcinoma (LUAD) cells^[16]^. Collectively, these observations indicate that diverse PTMs play critical roles in EGFR-TKI resistance and may represent promising therapeutic targets for overcoming it. In this review, we systematically explore how six key PTMs regulate EGFR. These modifications - phosphorylation^[17]^, palmitoylation^[18]^, ubiquitination^[19]^, glycosylation^[20]^, acetylation^[21]^, and S-nitrosylation^[22]^ - collectively influence EGFR’s subcellular localization, protein stability, and signaling dynamics. Based on this framework, we delineate four specific spatial resistance subtypes of EGFR: the degradation-evading^[23]^, membrane-retained^[24]^, nuclear^[25]^, and mitochondrial subtypes^[26]^. Each subtype exhibits a distinct molecular profile and differential susceptibility to treatment strategies^[13]^. Subsequently, this knowledge is synthesized into a PTM crosstalk network^[22,27]^. Under the guidance of the spatial fate model, we investigate how therapeutic targeting of this network may guide future strategies for TKI-resistant LC^[12,28]^.

## PHOSPHORYLATION OF EGFR

Phosphorylation, a reversible PTM catalyzed by protein kinases, involves the covalent attachment of a negatively charged and highly hydrophilic phosphate group to serine or tyrosine residues^[10]^. This modification induces conformational and functional changes in the target protein^[29]^. The activity of EGFR is precisely regulated by the opposing actions of kinases and phosphatases, which play critical roles in LC pathogenesis and contribute significantly to the development of TKI resistance^[10,30-32]^.

### Site-specific phosphorylation encodes diverse signaling outputs

**Figure 1:**
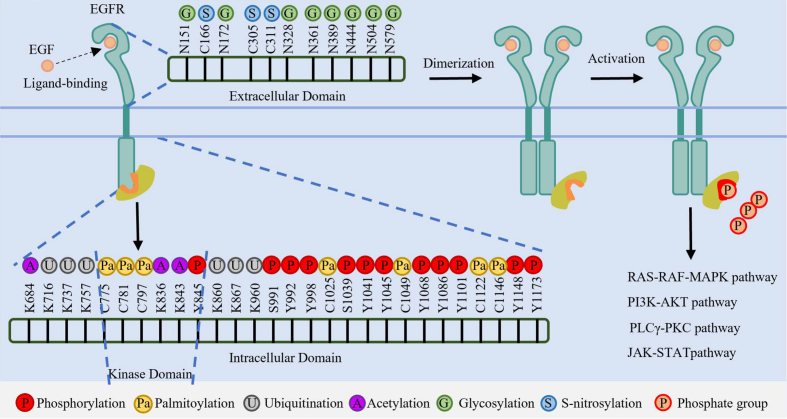
Schematic representation of PTMs on the EGFR extracellular and intracellular domains. Key modification sites, including Glycosylation (N-linked), Acetylation (K-linked), Ubiquitination (K-linked), Phosphorylation (Y/S-residues), Palmitoylation (C-residues), and S-nitrosylation (C-residues), are indicated. PTMs: Post-translational modifications; EGFR: epidermal growth factor receptor; N: asparagine; K: lysine; Y: tyrosine; S: serine; C: cysteine; EGF: epidermal growth factor; ATP: adenosine triphosphate; RAS: rat sarcoma viral oncogene homolog; RAF: rapidly accelerated fibrosarcoma; MAPK: mitogen-activated protein kinase; PI3K: phosphoinositide 3-kinase; AKT: protein kinase B; PLCγ: phospholipase C gamma; PKC: protein kinase C; JAK: Janus kinase; STAT: signal transducer and activator of transcription.

Upon EGF binding, EGFR undergoes dimerization and activates its intrinsic kinase domain, resulting in the phosphorylation of multiple tyrosine residues [Figure 1]. Key phosphorylated residues function as docking sites for downstream adaptors and effectors. For instance, phosphorylation at tyrosine (pY)1068 is recognized by growth factor receptor-bound protein 2 (Grb2), leading to activation of the rat sarcoma virus (RAS)-rapidly accelerated fibrosarcoma (RAF)-mitogen-activated protein kinase (MAPK) signaling pathway^[33-35]^. Src homology 2 domain-containing transforming protein (Shc) binds to pY1148 and pY1173^[36,37]^. Other phospho-sites mediate distinct interactions: pY1045 recruits Casitas B-lineage lymphoma proto-oncogene (CBL) to initiate receptor ubiquitination and degradation^[38,39]^; phospholipase C gamma (PLCγ) binds pY992 and pY1173^[40]^; Yes (Yamaguchi sarcoma viral oncogene homolog)/Lyn (Lck/Yes-related novel tyrosine kinase) associates with tyrosine (Y) 1101 to promote nuclear localization^[41]^; and phosphorylation at Y845 enhances Src binding, thereby amplifying proliferation signals^[27]^. This site-specific phosphorylation pattern enables precise regulation of several oncogenic pathways - such as RAS-RAF-MAPK, phosphatidylinositol 3-kinase (PI3K)-protein kinase B (AKT), PLCγ-protein kinase C (PKC), and Janus kinase (JAK)-signal transducer and activator of transcription (STAT) - which collectively drive tumor growth, migration, invasion, and angiogenesis in LC^[37]^. The key EGFR phosphorylation sites, their associated adaptors, and downstream pathways discussed above are summarized in Table 1.

**Table 1:** Phosphorylation sites of EGFR, associated signaling effectors, downstream pathways, and biological functions

**Phosphorylation site**	**Signaling effectors**	**Pathway**	**Function**	**Ref.**
Y845	Src	RAS/MAPK, PI3K/AKT	Cell proliferation, cell cycle control, mitochondrial regulation of cell metabolism and gamete activation	[27]
S991	Grb2/ CBL	RAS/ERK	Endocytic and trafficking	[17]
Y992	PLCγ	/	ROS level increase, ATP generation reduction, and apoptosis	[40]
Y998	Grb2/CBL	RAS/ERK	Endocytic and trafficking	[17]
S1039	/	p38 MAPK	Endocytic and trafficking	[17]
Y1041	/	p38 MAPK	Endocytic and trafficking	[17]
Y1045	CBL/Grb2	/	Ubiquitination and degradation	[38,39]
Y1068	Grb2/Shc	RAS/ERK	Proliferation, survival, and differentiation	[33-35]
Y1086	Grb2/Shc	RAS/ERK	Proliferation, survival, and differentiation	[33-35]
Y1101	SFKs	/	Nuclear localization, proliferation, survival, and migration	[41]
Y1148	Shc	RAS/ERK	Cell growth, survival, and metastasis	[36,37]
Y1173	Shc PLCγ	RAS/ERK	Cell growth, survival, and metastasis ROS Level Increase, ATP Generation Reduction, and Apoptosis	[36,37] [40]

EGFR: Epidermal growth factor receptor; Src: SRC proto-oncogene, non-receptor tyrosine kinase; Grb2: growth factor receptor-bound protein 2; CBL: Casitas B-lineage lymphoma proto-oncogene; PLCγ: phospholipase C gamma; Shc: Src homology 2 domain-containing transforming protein; SFKs: SRC family kinases; PI3K: phosphatidylinositol 3-Kinase; AKT: protein kinase B (PKB); p38 MAPK: p38 mitogen-activated protein kinase; RAS: rat sarcoma virus; RAF: rapidly accelerated fibrosarcoma; MAPK: mitogen-activated protein kinase.

### Phosphorylation directs EGFR subcellular trafficking and compartmentalized signaling

**Figure 2:**
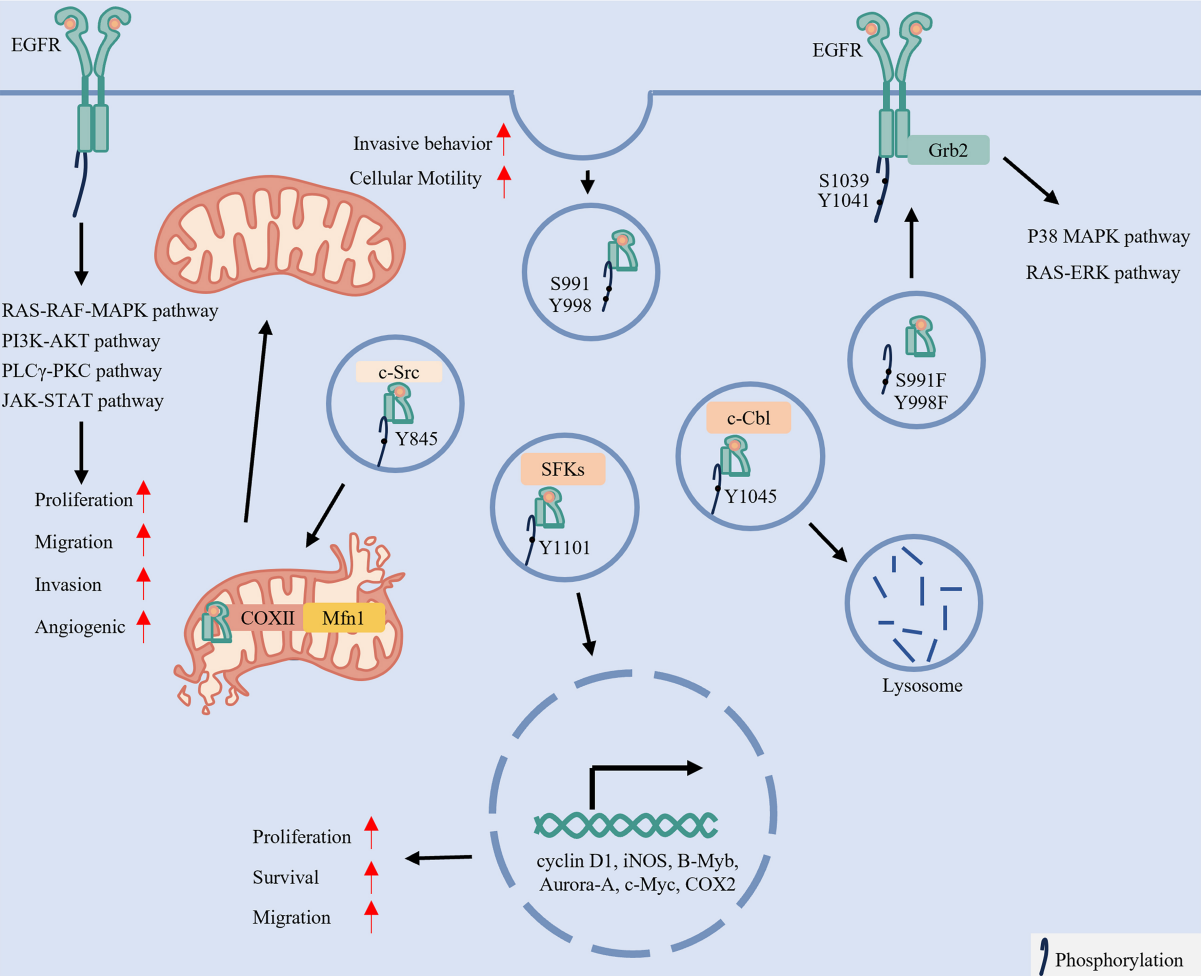
Site-specific phosphorylation of EGFR dictates its subcellular localization and oncogenic signaling. Upon ligand binding and dimerization, EGFR undergoes autophosphorylation, activating multiple oncogenic pathways such as the RAS-RAF-MAPK, PI3K-AKT, and JAK-STAT signaling, which drive cellular proliferation, migration, invasion and angiogenesis. Importantly, phosphorylation by specific kinases directs the trafficking of EGFR to distinct subcellular compartments. For instance, phosphorylation at serine 991, tyrosine 998, serine 1039, and tyrosine 1041 promotes its membrane localization or internalization. c-Src-mediated phosphorylation at Y845 facilitates mitochondrial localization, which induces mitochondrial fission and enhances cell motility. In contrast, SFK-mediated phosphorylation at Y1101 enables nuclear translocation, where EGFR functions as a transcriptional regulator. Meanwhile, CBL-mediated phosphorylation at Y1045 targets EGFR for lysosomal degradation. Created in BioRender. LIAN, J. (2026) https://BioRender.com/phddwmk. EGFR: Epidermal growth factor receptor; RAS: rat sarcoma virus; RAF: rapidly accelerated fibrosarcoma; MAPK: mitogen-activated protein kinase; PI3K: phosphatidylinositol 3-kinase; AKT: protein kinase B (PKB); JAK: Janus kinase; STAT: signal transducer and activator of transcription; SFKs: Src-family kinase; c-Cbl: Casitas B-lineage lymphoma proto-oncogene; Grb2: growth factor receptor-bound protein 2; PLCγ: phospholipase C gamma; PKC: protein kinase C; c-Src: SRC proto-oncogene, non-receptor tyrosine kinase; COX II: cytochrome c oxidase subunit II; Mfn1: Mitofusin 1; COX2: Cyclooxygenase-2; iNOS: inducible nitric oxide synthase; B-Myb: B-Myb transcription factor; Aurora-A: aurora kinase A; c-Myc: MYC proto-oncogene protein.

In addition to mediating membrane signaling, EGFR phosphorylation plays a critical role in receptor internalization and subsequent trafficking^[17,35]^. As summarized in Figure 2, site-specific phosphorylation serves as a molecular address code that directs EGFR into one of three main trafficking pathways: endocytic^[17,35]^, nuclear^[41]^ or mitochondrial^[27]^.

Endocytosis and Signaling: Phosphorylation at serine 991 and tyrosine 998 promotes clathrin-mediated endocytosis. Mutations in this region elevate phosphorylation at serine 1039 and tyrosine 1041, facilitating Grb2 recruitment^[17,35]^. Consequently, EGFR internalization is enhanced, along with increased activation of downstream RAS-ERK and p38 MAPK pathways^[17,35]^. In summary, EGFR signaling is not a simple binary switch but rather a finely regulated code, encrypted through site-specific phosphorylation.

Nuclear Translocation and Transcriptional Regulation: EGFR nuclear translocation initiates following endocytosis. This process is promoted by phosphorylation at Y1101 mediated by SRC Family Kinases (SFKs), particularly Yes and Lyn^[41]^. Within the nucleus, EGFR acts as both a transcriptional co-regulator and a kinase, modulating the expression of genes such as cyclin D1, inducible nitric oxide synthase (iNOS), and MYC proto-oncogene (MYC), thereby influencing oncogenic growth, survival, and cell migration^[42]^.

Mitochondrial Localization and Metabolic Rewiring: Src-mediated phosphorylation at Y845 recruits cytochrome c oxidase subunit II (CoxII), directing a subpopulation of EGFR to mitochondria^[43-45]^. Within mitochondria, EGFR interacts with and destabilizes the fusion protein Mitofusin 1 (Mfn1), thereby promoting mitochondrial fission and facilitating the redistribution of mitochondria to the leading edge of cells. This process subsequently enhances the migratory and invasive capacities of LC cells^[46]^.

Thus, site-specific phosphorylation directs EGFR to distinct subcellular destinations, underpinning its diverse roles in LC.

### A spatial phosphorylation model: understanding and overcoming TKI resistance

Phosphorylation at specific tyrosine residues dictates the subcellular localization and functional role of EGFR. In light of this mechanism and the central challenge of TKI resistance, we classify resistance into distinct subtypes based on these differential intracellular localizations^[47]^.

Degradation-evading Subtype: Inhibition of Y1045 phosphorylation by TKIs impairs recruitment of the E3 ligase CBL, disrupting ubiquitin-mediated lysosomal degradation. This results in enhanced EGFR recycling, prolonged membrane retention, and sustained pro-survival signaling - a phenotype termed the “degradation-evading subtype”^[13,48]^. Notably, combining T315 with TKIs has been shown to overcome such resistance^[13]^, offering a targeted strategy to restore lysosomal degradation in this resistance subtype.

The Nuclear and Mitochondrial Subtypes: Conventional TKIs do not inhibit alternative kinases such as SFKs^[49]^. Consequently, SFK-mediated phosphorylation at Y1101 (by Yes/Lyn) and Src-mediated phosphorylation at Y845 can drive the development of the nuclear and mitochondrial subtypes, respectively^[50]^. These subtypes sustain pro-survival and proliferative signaling through pathways independent of canonical membrane-bound EGFR kinase activity^[49]^. Therefore, targeting SFKs/Src with inhibitors such as dasatinib represents a potential strategy to overcome nuclear- and mitochondrial-mediated resistance in LC^[51]^.

## PALMITOYLATION OF EGFR

Palmitoylation is a reversible lipid modification involving the attachment of palmitate to specific cysteine (Cys) residues, catalyzed by DHHC (Asp-His-His-Cys) family acyltransferases and reversed by depalmitoylases^[52-54]^. This dynamic PTM regulates protein trafficking^[53]^, stability^[55]^, and signaling^[56]^, and has been increasingly implicated in cancer pathogenesis and TKI resistance in LC^[57,58]^.

### Site-specific palmitoylation governs EGFR signaling output

EGFR palmitoylation occurs at multiple Cys residues primarily within its intracellular domains, with key modification sites identified as Cys1025, Cys1034, and Cys1122 in the C-terminal tail, as well as Cys797 in the kinase domain, and additional sites including Cys775, Cys818, Cys939, Cys950, Cys1049, Cys1058, and Cys1146^[18,24-26]^. These palmitoylation events are primarily catalyzed by DHHC20 and DHHC13, with DHHC13 specifically regulating the palmitoylation of Cys775, Cys781, and Cys797 sites in EGFR^[18]^; however, the modification sites modulated by DHHC20 remain unclear [Table 2]. Functionally, DHHC20 knockdown suppresses PI3K/AKT signaling while enhancing MAPK activity, altering the activity of oncogenic transcription factors such as MYC. This shift sensitizes cells to PI3K inhibitors and inhibits tumor growth in Kirsten Rat Sarcoma viral oncogene homolog (KRAS)-mutant LC models^[59]^.

**Table 2:** Palmitoylation sites of EGFR, corresponding palmitoyltransferases, subcellular localization, and biological functions

**Palmitoylation site**	**Palmitoyltransferase**	**Localization of EGFR**	**Function**	**Ref.**
C775	DHHC13	Membrane localization	/	[18]
C781	DHHC13	Membrane localization	/	[18]
C797	DHHC13	Mitochondrial/Nuclear/Membrane localization	Cellular metabolism, survival, proliferation	[18,25,26]
C1025	/	Membrane localization	Cell migration, transformation	[24]
C1049	/	Nuclear localization	Proliferation and tumor growth	[25]
C1122	/	Internalization	Cell migration, transformation, degradation	[24]
C1146	/	Nuclear localization	Proliferation and tumor growth	[25]

EGFR: Epidermal growth factor receptor; DHHC13: DHHC-type palmitoyltransferase 13.

### Palmitoylation directs EGFR subcellular trafficking and compartment-specific functions

**Figure 3:**
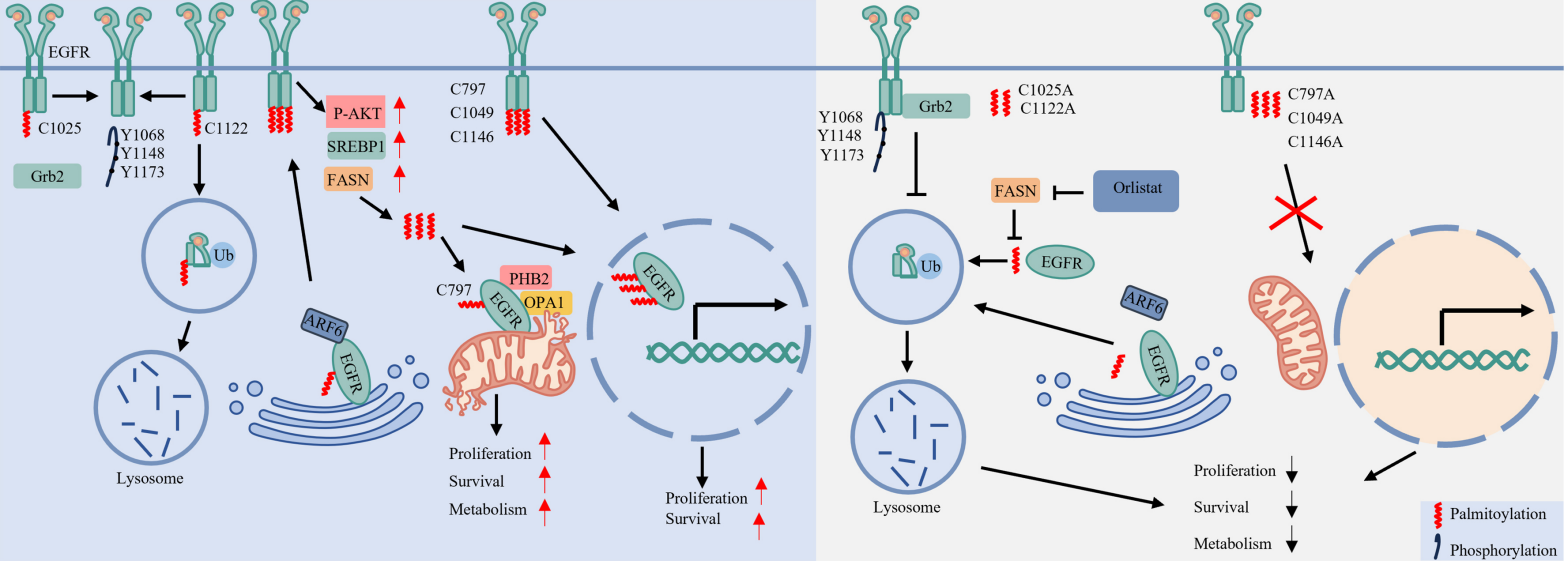
Palmitoylation coordinates EGFR membrane dynamics and organelle-specific signaling. Catalyzed by DHHC-family palmitoyltransferases (such as DHHC13 and DHHC20), palmitoylation at specific cysteine residues critically regulates EGFR subcellular localization. Palmitoylation anchors EGFR to the plasma membrane through the ARF6-dependent sorting pathway. However, palmitoylation at different cysteines elicits distinct functional outcomes: palmitoylation at C1025 inhibits Grb2 binding and downstream signaling, whereas palmitoylation at C1122 promotes endocytosis and subsequent lysosomal degradation. Meanwhile, FASN-mediated palmitoylation at C797 facilitates both mitochondrial translocation - enhancing mitochondrial fusion and cell survival via the PHB2/OPA1 axis - and nuclear import, thereby coordinating metabolic adaptation and proliferation. Created in BioRender. LIAN, J. (2026) https://BioRender.com/ivso7d7. EGFR: Epidermal growth factor receptor; ARF6: ADP-ribosylation factor 6; Grb2: growth factor receptor-bound protein 2; FASN: Fatty Acid Synthase; PHB2: prohibitin 2; OPA1: optic atrophy 1; P-AKT: phosphorylated protein kinase B; SREBP1: sterol regulatory element-binding protein 1.

Beyond its role in signal modulation, palmitoylation also governs the trafficking of EGFR to distinct organelles. Acting as a spatial trafficking motif, it directs the receptor to specific subcellular destinations [Figure 3].

Membrane Trafficking: Palmitoylation enables EGFR to engage the ADP-ribosylation factor 6 (ARF6)-dependent sorting machinery for efficient transport from the Golgi to the plasma membrane. Loss of palmitoylation disrupts this trafficking, resulting in intracellular receptor accumulation^[18]^. Conversely, palmitoylation at Cys1025 anchors the unstructured C-terminal tail to the plasma membrane, which inhibits Grb2 binding and subsequently suppresses phosphorylation at Y1068, Y1148, and Y1173^[24]^.

Internalization and Degradation: Palmitoylation further regulates EGFR intracellular trafficking. Upon EGF stimulation, palmitoylation at Cys1122 facilitates receptor internalization and subsequent lysosomal targeting for degradation^[24]^. In contrast, cells expressing a Cys1122 mutant exhibit disrupted trafficking: internalized EGFR vesicles lack early and late endosomal markers and fail to undergo recycling, indicating a near-complete trafficking arrest^[24]^. Notably, these stalled vesicles sustain prolonged EGFR phosphorylation and persistent AKT signaling.

Mitochondrial Localization and Metabolic Survival: Palmitoylation initiates a feed-forward cycle by activating AKT, which upregulates the lipogenic transcription factor Sterol regulatory element-binding protein 1 (SREBP1) and its downstream target FASN (fatty acid synthase)^[25]^. FASN-generated palmitate subsequently promotes palmitoylation of mitochondrial EGFR at Cys797^[60]^. This modification stabilizes mitochondrial EGFR, enhances mitochondrial fusion via upregulation of Prohibitin 2 (PHB2) and Optic Atrophy 1 (OPA1), and supports cell survival - thus connecting lipid metabolism to proliferative adaptation^[25,60]^.

Nuclear Localization: FASN supplies palmitate for EGFR palmitoylation, which at residues Cys797, Cys1049, and Cys1146 is essential for receptor nuclear translocation^[25]^. This process sustains a nuclear EGFR pool that drives proliferation and tumorigenesis in EGFR-mutant NSCLC cells^[61]^. Consistent with this mechanism, mutation of these cysteine residues abolishes nuclear import and inhibits cell proliferation, without altering EGFR membrane localization^[25,61]^.

In summary, site-specific palmitoylation directs EGFR to distinct subcellular compartments - including the plasma membrane, lysosome, mitochondria, and nucleus - thereby defining its compartment-specific functions in LC.

### A spatial palmitoylation model for understanding and overcoming TKI resistance

Site-specific palmitoylation determines EGFR subcellular localization and modulates its signaling functions. Based on these distinct localization patterns, we have defined several resistance subtypes to address TKI resistance in LC.

Membrane-Retained Subtype: DHHC20-mediated palmitoylation at Cys1025 and Cys1122 enhances Grb2 binding and EGFR phosphorylation, thereby sensitizing cells to EGFR inhibitors^[24]^. Potential DHHC20 inhibitors include natural compounds such as lutein, 5-hydroxyflavone, and 6-hydroxyflavone, which demonstrate high predicted binding affinity and stability in silico, supporting their further development as low-toxicity agents^[62]^. Additionally, strategies such as disrupting the ARF6-dependent trafficking pathway - for example, with the N-myristoylated Glycine-Lysine-Valine-Leucine Trans-Activator Transcription protein (GKVL-TAT) peptide - represent viable approaches to suppress EGFR membrane localization^[18]^.

The Mitochondrial and Nuclear Subtypes: Palmitate produced by FASN drives EGFR palmitoylation at Cys797^[43]^. This modification supports metabolic survival in the mitochondrial subtype and facilitates transcriptional reprogramming in the nuclear subtype^[43,60]^. Both subtypes are pharmacologically susceptible to FASN inhibition (e.g., with orlistat), which reduces palmitate availability, impairs organellar trafficking, and suppresses tumor progression^[25,61,63]^.

## UBIQUITINATION OF EGFR

Ubiquitination is a reversible, covalent modification in which ubiquitin molecules are attached to lysine (K) residues^[64]^. This process is mediated by an E1-E2-E3 enzymatic cascade and reversed by deubiquitinating enzymes (DUBs)^[65]^. By regulating EGFR levels, localization, signaling, and protein interactions, ubiquitination plays a critical role in LC pathogenesis and therapeutic response^[64-66]^.

### Site-specific ubiquitination controls EGFR signaling and degradation

**Figure 4:**
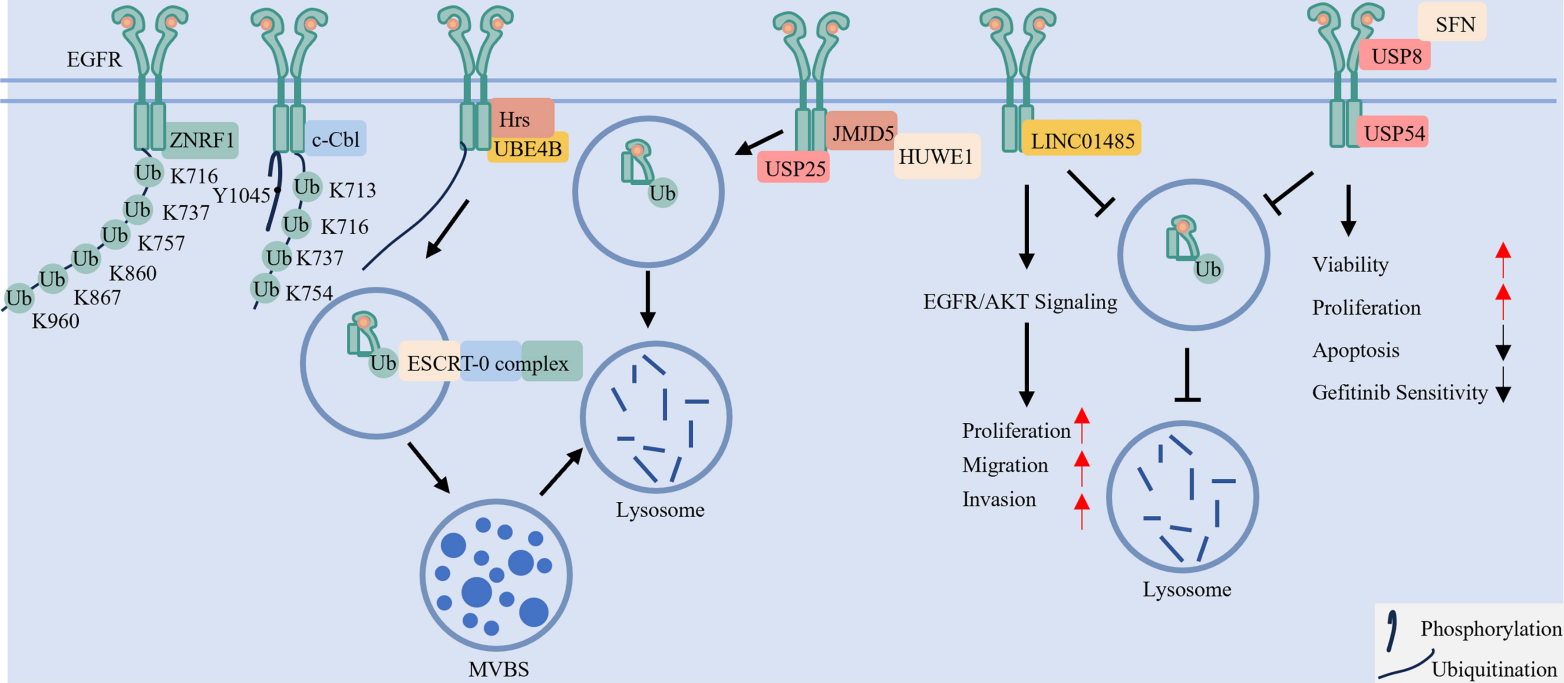
The ubiquitination-deubiquitination axis controls EGFR endocytic sorting, degradation, and recycling. Ligand-activated EGFR is ubiquitinated at specific lysine residues by E3 ligases such as c-Cbl and ZNRF1. This ubiquitin tag is recognized by endocytic machinery (e.g., Hrs, UBE4B) and the ESCRT complex, sorting EGFR into intraluminal vesicles of MVBs for ultimate lysosomal degradation and signal termination. The process is dynamically opposed by DUBs such as USP8 and USP54, which stabilize EGFR and can promote its recycling to the plasma membrane for signal reactivation. Conversely, USP25 and HUWE1 (with the help of JMJD5) can promote the degradation of EGFR. EGFR: Epidermal growth factor receptor; c-Cbl: Casitas B-lineage lymphoma proto-oncogene; ZNRF1: Zinc and Ring Finger 1; Hrs: hepatocyte growth factor-regulated tyrosine kinase substrate; UBE4B: Ubiquitination Factor E4B; ESCRT: endosomal sorting complex required for transport; MVBs: multivesicular bodies; DUBs: deubiquitinating enzymes; USP: Ubiquitin-Specific Peptidase; HUWE1: HECT, UBA and WWE Domain Containing E3 Ubiquitin Protein Ligase 1; JMJD5: Jumonji Domain Containing 5; SFN: Stratifin.

EGFR ubiquitination primarily occurs on lysine residues within its tyrosine kinase domain, with specificity conferred by distinct E3 ligases [Figure 4]. For example, CBL targets residues such as K713 and K737, whereas Zinc and Ring Finger 1 (ZNRF1) modifies a different set including K716 and K960^[19]^. Ligand-induced autophosphorylation at Y1045 creates a docking site for CBL, tightly coupling receptor activation with subsequent ubiquitination^[48]^. CBL-mediated ubiquitination then directs EGFR toward endocytosis and lysosomal degradation, attenuating downstream signaling^[48,67]^. Thus, phosphorylation serves as a molecular switch that initiates ubiquitination-dependent signal termination, preventing excessive EGFR activation.

### A dynamic ubiquitination/deubiquitination network determines EGFR fate

The ubiquitination state of EGFR is dynamically regulated by the opposing activities of E3 ligases and DUBs, which collectively determine receptor stability and function^[68,69]^. For example, HUWE1 (HECT, UBA and WWE Domain Containing E3 Ubiquitin Protein Ligase 1), assisted by JMJD5 (Jumonji Domain Containing 5), promotes proteasomal degradation^[70,71]^. In contrast, certain DUBs stabilize EGFR: Ubiquitin-Specific Peptidase (USP)54 and the USP8/Stratifin (SFN) complex support NSCLC cell growth and survival by protecting EGFR from degradation^[72,73]^. Conversely, USP25 facilitates degradation by activating CBL^[74]^. This regulatory network also involves non-coding RNAs, such as long intergenic non-protein coding RNA 1485 (LINC01485), which suppresses EGFR ubiquitination to activate EGFR/AKT signaling and drive tumorigenesis^[75]^. Together, these components constitute a multifaceted ubiquitin-based regulatory circuit that finely tunes EGFR signaling.

### Ubiquitination directs EGFR subcellular trafficking and localization

Following ligand binding, recruited E3 ligases ubiquitinate activated EGFR, a modification that directly governs subsequent receptor trafficking and fate^[76,77]^. The topology of ubiquitin chains - whether mono- or polyubiquitination - specifies the endocytic route, such as clathrin-dependent or -independent pathways^[76]^. Ubiquitinated EGFR then recruits endocytic adaptors with ubiquitin-binding domains [e.g., Ubiquitination Factor E4B (UBE4B), Hepatocyte growth factor-Regulated Tyrosine kinase Substrate (Hrs)] to mediate its entry into endosomal compartments^[78]^. Within endosomes, the Endosomal Sorting Complex Required for Transport (ESCRT) complex recognizes ubiquitinated EGFR and packages it into intraluminal vesicles of multivesicular bodies (MVBs)^[79]^. These MVBs are subsequently delivered to lysosomes for degradation, a process whose efficiency correlates with the extent of ubiquitination^[19]^. Notably, not all ubiquitinated EGFR is degraded; a portion can be deubiquitinated and recycled to the plasma membrane, enabling signal reactivation^[80]^. The key enzymes and regulators involved in these processes are summarized in Table 3. Collectively, ubiquitination finely regulates EGFR signaling output by coordinating its endocytic itinerary, balancing degradation with recycling.

**Table 3:** The enzyme, interaction protein and function of EGFR

**Enzyme**	**Interaction protein**	**Function**	**Ref.**
USP8	SFN	Stabilizing EGFR	[73]
USP54	/	Stabilizing EGFR	[72]
/	LINC01485	Stabilizing EGFR	[75]
USP25	CBL	Enhancing the endocytosis and degradation of EGFR	[74]
USP8	STAM	Promoting the endosomal sorting and lysosomal degradation of EGFR	[78]
UBE4B	Hrs and ESCRT-0 complex	Associating with endosomal membranes and ensuring its proper trafficking into the endosomal system	[78]
CBL	/	Being recruited to EGFR at the membrane, catalyzing its ubiquitination	[77]
HUWE1	JMJD5	Enhancing EGFR proteasomal degradation	[70]

EGFR: Epidermal growth factor receptor; USP: Ubiquitin-Specific Peptidase; SFN: Stratifin; CBL: Casitas B-lineage lymphoma proto-oncogene; STAM: signal-transducing adaptor molecule; UBE4B: Ubiquitination Factor E4B; Hrs: hepatocyte growth factor-regulated tyrosine kinase substrate; ESCRT: endosomal sorting complex required for transport; HUWE1: HECT, UBA and WWE Domain Containing E3 Ubiquitin Protein Ligase 1; JMJD5: Jumonji Domain Containing 5.

### Spatial ubiquitination model of therapy against TKI resistance

Studies indicate that enhancing EGFR ubiquitination promotes its degradation, thereby suppressing oncogenic signaling in TKI-resistant LC^[69,72]^. Impairment of this regulatory checkpoint underlies the “degradation-evading” resistance mechanism, which sustains oncogenic signaling despite treatment^[72]^. Consequently, therapeutic strategies aimed at restoring or augmenting EGFR ubiquitination may facilitate receptor degradation^[72]^. For example, smallmolecule inhibitors targeting the DUB USP8 can reduce EGFR levels in TKIresistant NSCLC models by accelerating receptor turnover and inducing apoptosis^[81]^. Additionally, PROTACs, which exploit the ubiquitinproteasome system to degrade specific proteins, represent another promising therapeutic avenue^[14]^.

## GLYCOSYLATION OF EGFR

Glycosylation is an enzymatic process that attaches carbohydrate moieties to proteins, influencing their folding, stability, localization, and interactions^[50,82]^. As a key post-translational modification of EGFR, glycosylation critically regulates receptor activity and contributes to LC pathogenesis and TKI resistance^[16,83]^.

### Site-specific glycosylation encodes bidirectional regulation of EGFR signaling

EGFR undergoes extensive N-glycosylation at specific asparagine residues (e.g., N361, N444), a modification essential for receptor activation as it enhances ligand binding and dimerization^[20]^. Glycans attached at specific positions - such as N151, N172, N328, N389, and N504 - lift the receptor’s structure above the membrane and prevent critical ligand-binding domains from being too close to the lipid bilayer^[84]^. In contrast, sialylation at sites such as N32, N151, and N389 inhibits EGFR by sterically hindering ligand interaction and blocking autophosphorylation of key tyrosine residues (e.g., Y1068, Y1086, Y1173)^[85]^. Consequently, mutation of critical glycosylation sites (e.g., N361, N444, N579) reduces ligand sensitivity, attenuates downstream signaling, and impairs cancer cell growth^[20,50,86]^.

A network of glycosyltransferases exerts opposing effects on EGFR signaling. For example, core fucosylation by fucosyltransferase 8 (FUT8) promotes receptor activation, whereas FUT4/FUT6 may oppose this effect. Similarly, ST6 Beta-Galactoside Alpha-2,6-Sialyltransferase 1 (ST6GAL1) enhances EGFR-driven signaling via the AKT/nuclear factor κB (NF-κB) pathway^[87,88]^.

**Figure 5:**
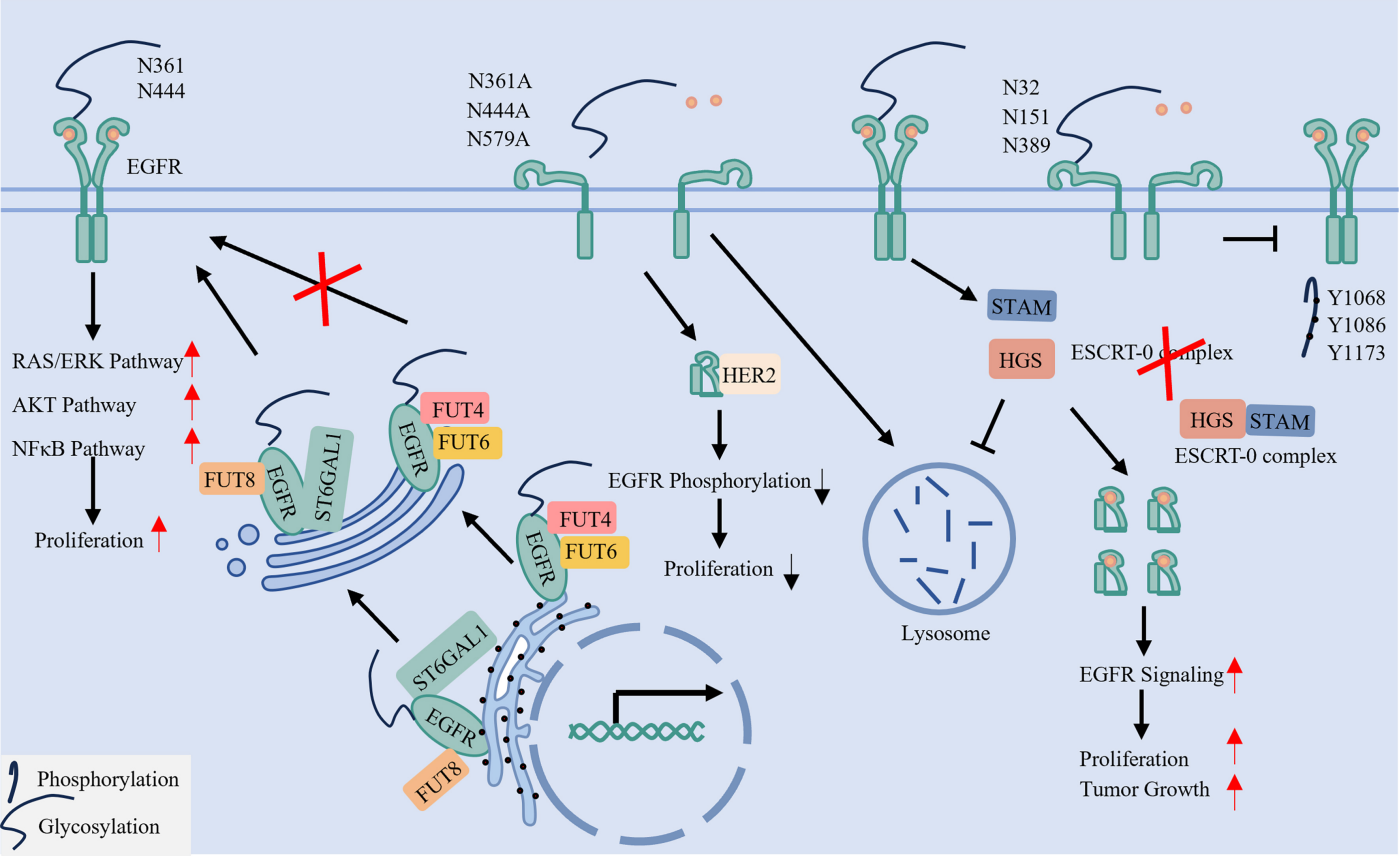
N-glycosylation ensures proper EGFR conformation, membrane positioning, and stability. N-glycosylation in the endoplasmic reticulum and Golgi apparatus, mediated by glycosyltransferases (e.g., FUT8, ST6GAL1), is essential for EGFR function. Glycans attached at specific sites (e.g., N361, N444) lift the extracellular domain for proper ligand binding and dimerization. In contrast, sialylation at other sites (e.g., N32, N151) can inhibit activation. Furthermore, glycosylation, particularly O-GlcNAcylation of the ESCRT-0 component HGS, can impede receptor degradation, thereby stabilizing EGFR. EGFR: Epidermal Growth Factor Receptor; FUT: Fucosyltransferase; ST6GAL1: ST6 Beta-Galactoside Alpha-2,6-Sialyltransferase 1; O-GlcNAcylation: O-linked β-N-acetylglucosaminylation; ESCRT: Endosomal Sorting Complex Required for Transport; HGS: Hepatocyte growth factor-regulated tyrosine kinase substrate. EGFR: Epidermal growth factor receptor; FUT: fucosyltransferase; O-GlcNAcylation: O-linked β-N-acetylglucosaminylation; ESCRT: endosomal sorting complex required for transport; HGS: hepatocyte growth factor-regulated tyrosine kinase substrate; STAM: signal-transducing adaptor molecule; RAS: rat sarcoma virus; ERK: extracellular signal-regulated kinase; AKT: protein kinase B; NF-κB: nuclear factor kappa-light-chain-enhancer of activated B cells.

Thus, signaling output is precisely regulated through specific enzyme-site pairings, enabling bidirectional modulation. These residues, associated enzymes, and functional outcomes are summarized in Table 4 and illustrated in Figure 5.

**Table 4:** The glycosylation site, enzyme and function of EGFR

**Glycosylation site**	**Enzyme**	**Function**	**Ref.**
N151/N172/N328/N389/N504	/	Lifting the receptor’s structure above the membrane and preventing critical ligand-binding domains from being too close to the lipid bilayer	[84]
N32/N151/N389	/	Hindering ligand interaction and blocking autophosphorylation	[85]
N361/N444/N579	/	Promoting EGF binding and the dimerization, activation of EGFR	[20,86]
/	FUT8/ST6GAL1	Promoting EGF binding and the dimerization, activation of EGFR	[87,88]
/	FUT4/FUT6	Inhibiting EGF binding and the dimerization, activation of EGFR	[87]

EGFR: Epidermal growth factor receptor; FUT: fucosyltransferase; ST6GAL1: ST6 Beta-Galactoside Alpha-2,6-Sialyltransferase 1; EGF: epidermal growth factor.

### Glycosylation directs EGFR membrane localization and structural integrity

Glycosylation regulates EGFR membrane organization and structural integrity, as illustrated in Figure 5.

Orientation and Activation: Glycosylation critically regulates EGFR structural integrity, membrane orientation, and functional activation. Site-specific glycans elevate the receptor extracellular domain away from the lipid bilayer, positioning ligand-binding regions for optimal interaction and facilitating subsequent receptor activation^[20,84,89]^.

Protein Conformation and Trafficking Stability: Glycosylation stabilizes the EGFR extracellular domain by preventing misfolding and maintaining its structural integrity^[90,91]^. Additionally, O-linked β-N-acetylglucosaminylation (O-GlcNAcylation) impairs the hepatocyte growth factor-regulated tyrosine kinase substrate (HGS)-signal-transducing adaptor molecule (STAM) interaction, which disrupts ESCRT-0 complex assembly and function, thereby suppressing EGFR lysosomal degradation^[92]^.

In summary, glycosylation critically regulates both the structural integrity and degradation dynamics of EGFR, underscoring its role as a multifaceted functional modulator.

### Targeting glycosylation as a therapeutic strategy in TKI resistance

Glycosylation contributes significantly to TKI resistance in LC^[16]^. EGFR function is finely tuned through site-specific glycosylation, executed by dedicated glycosyltransferases that regulate distinct receptor activities^[20]^. Based on this, we define two glycosylation-dependent resistance subtypes: a membrane-retained subtype, where altered glycosylation stabilizes EGFR at the plasma membrane^[20]^, and a degradation-evading subtype, in which glycosylation disrupts lysosomal trafficking^[92]^. The specific glycosyltransferases responsible for the latter subtype, however, have yet to be identified.

Current therapeutic strategies targeting glycosylation focus on inhibiting specific pathway enzymes. For the membrane-retained subtype, NGI-1 (an inhibitor of N-glycosylation initiation) and 3Fax-Peracetyl Neu5Ac (a ST6GAL1 inhibitor) can suppress EGFR activation and downstream signaling^[93,94]^. Notably, the conventional chemotherapeutic agent cisplatin also functions as a FUT8 fucosyltransferase inhibitor^[95-97]^. Combining such glycosylation inhibitors with TKIs may therefore provide an effective approach against this resistance subtype.

## ACETYLATION OF EGFR

Acetylation, a reversible post-translational modification involving the addition of an acetyl group to lysine residues, is dynamically regulated by acetyltransferases and deacetylases^[98]^. This modification influences protein function by altering charge, conformation, and interaction networks^[98]^. For EGFR, acetylation plays a critical role in modulating its activity, intracellular trafficking, and downstream signaling, thereby contributing to tumor progression and cancer cell survival in TKI-resistant LC^[98-100]^.

### Site-specific acetylation dynamics

**Figure 6:**
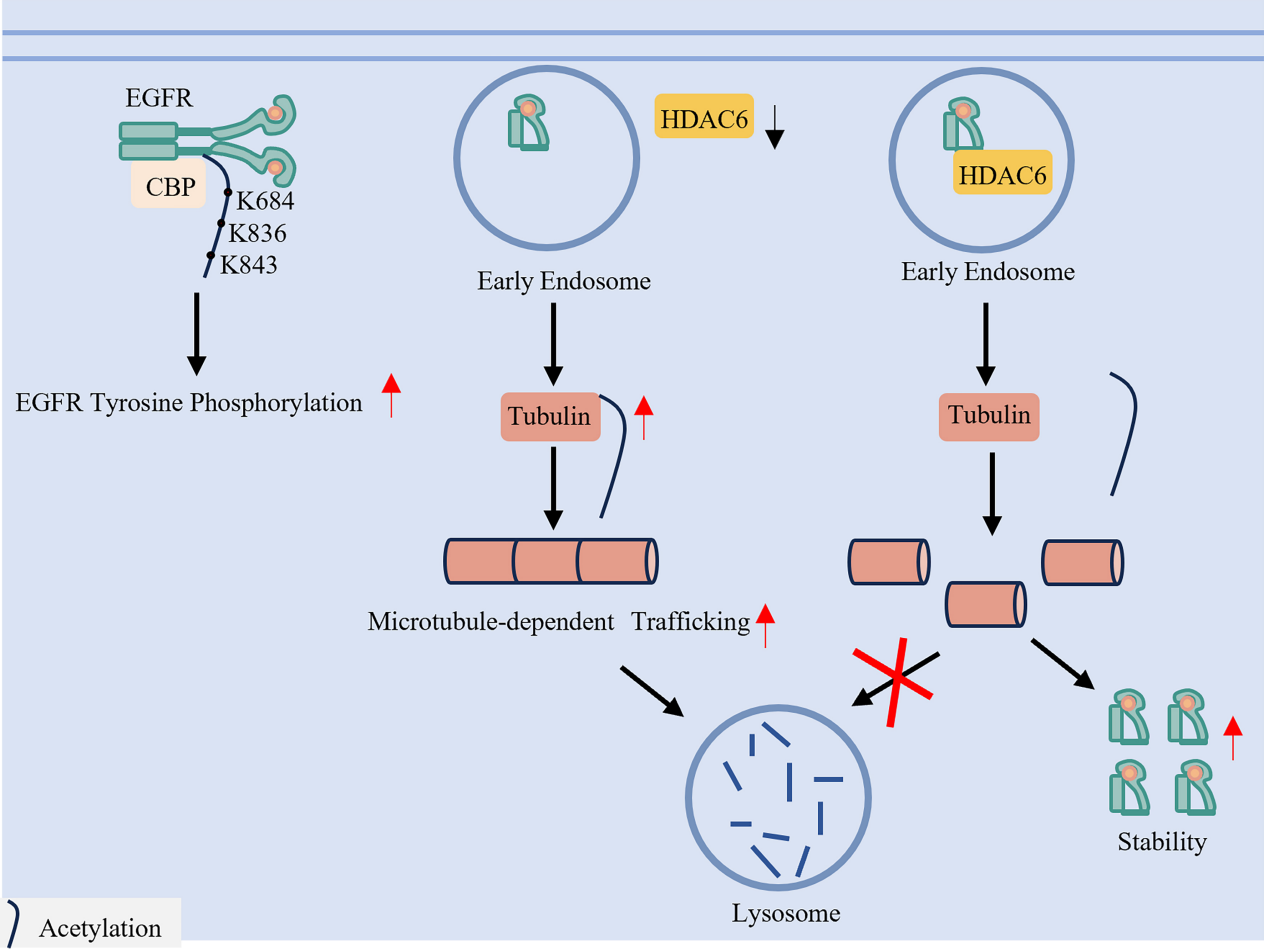
Acetylation and deacetylation fine-tune EGFR trafficking and degradation. Acetylation of EGFR by acetyltransferases such as CBP enhances receptor phosphorylation and signaling. A key regulatory mechanism involves HDAC6, a deacetylase that acts on α-tubulin. HDAC6-mediated deacetylation of microtubules delays the trafficking of EGFR from early endosomes to late endosomes/lysosomes, thereby impeding receptor degradation and potentially sustaining signaling. EGFR: Epidermal growth factor receptor; CBP: CREB-binding protein; HDAC6: histone deacetylase 6.

It is reported that CBP [cyclic adenosine monophosphate (cAMP)-responsive element-binding protein (CREB)-binding protein] is the primary acetyltransferase responsible for EGFR acetylation at K684, K836, and K843 residues, with its endocytosis occurring upon EGF stimulation and thereby contributing to enhanced EGFR phosphorylation^[101]^. On the deacetylation side, histone deacetylase 6 (HDAC6) regulates EGFR turnover through deacetylation, and its inhibition not only increases α-tubulin acetylation but also decreases the phosphorylation of EGFR/AKT signaling and induces cell survival and proliferation^[21]^. The site-specific acetylation patterns mediated by different enzymes are summarized in Table 5 and illustrated in Figure 6.

**Table 5:** The enzyme, modification sites, function of EGFR acetylation

**Modification types**	**Enzyme**	**Modification sites**	**Function**	**Ref.**
Acetylation	CBP	K684/K836/K843	EGF stimulation and EGFR phosphorylation	[101]
	HDAC6	/	Delaying EGFR transport from early to late endosomes by deacetylating α-tubulin, thereby impeding microtubule-dependent trafficking toward lysosomal degradation	[21]

EGFR: Epidermal growth factor receptor; CBP: CREB-binding protein; HDAC6: Histone deacetylase 6; EGF: epidermal growth factor.

Upregulation of HDAC6 deacetylates α-tubulin, impairing microtubule-dependent trafficking of EGFR from early to late endosomes and thereby blocking its lysosomal degradation^[102]^. This mechanism underlies a “degradation-evading” resistance subtype, in which impaired receptor turnover sustains oncogenic signaling despite TKI treatment.

### Therapeutic targeting of the acetylation/deacetylation axis

Targeting acetylation offers a promising approach to overcome TKI resistance in LC. Preclinical evidence indicates that HDAC6 inhibition can restore sensitivity to EGFR-TKIs^[103]^. Several HDAC6 inhibitors, including ricolinostat (ACY)-1215, ACY-241, KA2507, and JBI-802, are currently in Phase 1 or Phase 2 clinical trials^[104,105]^. Additionally, FT-6876, a potent CBP bromodomain inhibitor, suppresses cancer cell proliferation and represents a potential therapeutic strategy for TKI-resistant LC^[106]^.

## S-NITROSYLATION OF EGFR

S-Nitrosylation is a nitric oxide (NO)-dependent, reversible post-translational modification in which NO forms S-nitrosothiols with cysteine thiols. This modification alters protein structure, function, and interactions, thereby regulating cellular signaling and contributing to disease pathogenesis^[22]^. In the case of EGFR, S-nitrosylation critically modulates its activity and downstream oncogenic signaling, promoting tumor progression and therapy resistance^[22,107,108]^.

### Site-specific S-nitrosylation dichotomously regulates EGFR function and membrane localization

**Figure 7:**
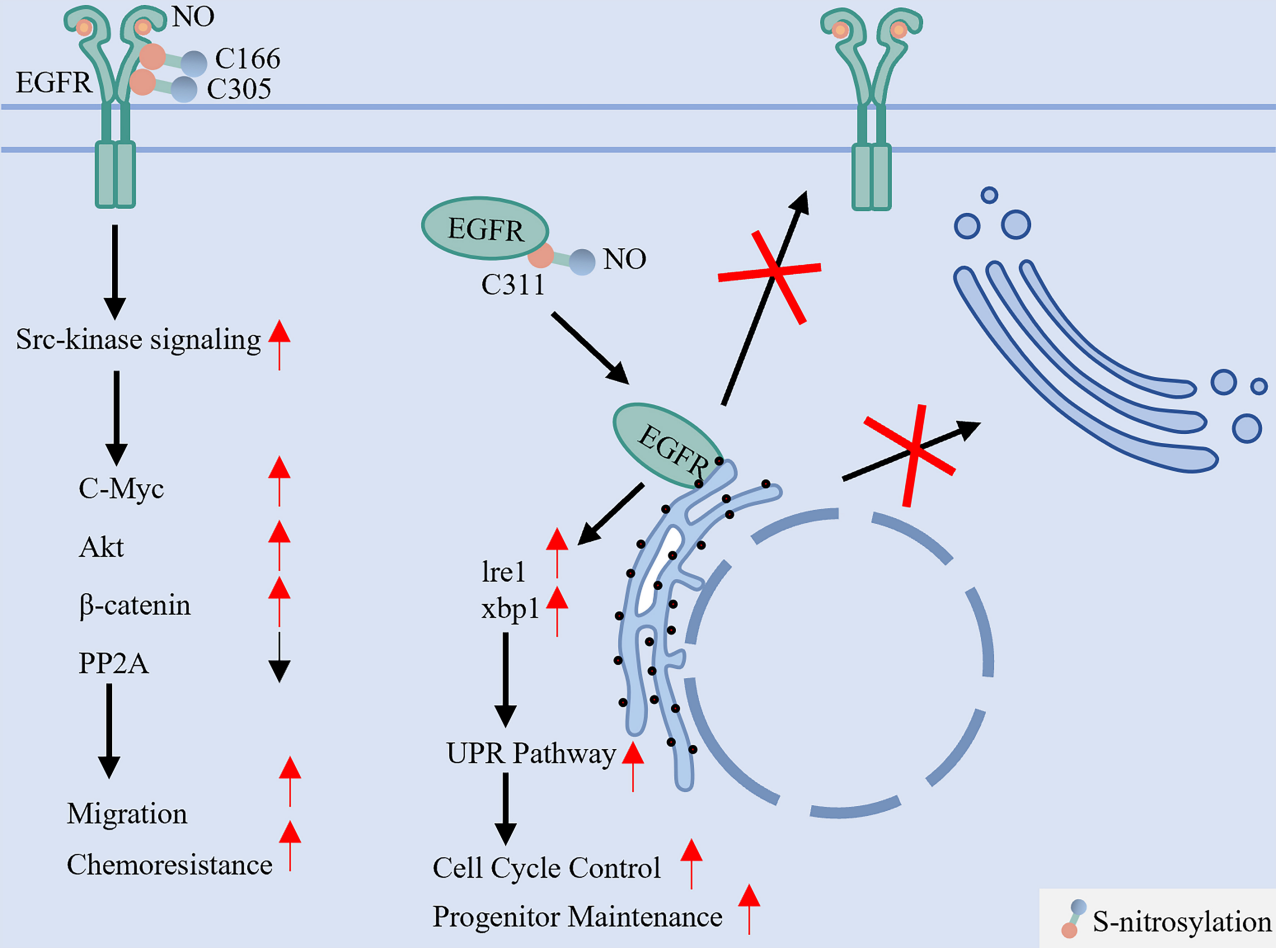
S-nitrosylation dichotomously regulates EGFR activation and maturation. This reversible modification, mediated by NO derived from NOS2, targets specific cysteine residues with opposing outcomes. S-nitrosylation at C166 and C305 enhances EGFR interaction with Src, activating downstream oncogenic pathways (e.g., Akt, MYC) that promote cell migration and chemoresistance. In stark contrast, S-nitrosylation at the extracellular C311 residue disrupts native disulfide bond formation, leading to protein misfolding, ER retention, and impaired membrane localization. EGFR: Epidermal growth factor receptor; NO: nitric oxide; NOS2: nitric oxide synthase 2; Src: SRC proto-oncogene, non-receptor tyrosine kinase; Akt: protein kinase B (PKB); MYC: MYC proto-oncogene; ER: endoplasmic reticulum; PP2A: protein phosphatase 2A; Ire1: inositol-requiring enzyme 1; xbp1: X-box binding protein 1; UPR: unfolded protein response.

S-Nitrosylation of EGFR exerts opposing functional and conformational effects depending on the modified cysteine residue [Figure 7]. Two key mechanisms are highlighted:

(1) Nitrosylation at Cys166 and Cys305 - mediated by nitric oxide synthase 2 (NOS2)-derived NO - enhances downstream signaling via Src kinase activation, stimulating oncogenic pathways such as MYC, AKT, and β-catenin to promote cell migration and chemoresistance^[22,109]^; (2) Conversely, S-nitrosylation at Cys311 disrupts native disulfide bonds in the extracellular domain, resulting in misfolding, endoplasmic reticulum retention, and impaired plasma membrane delivery^[110]^. Key S-nitrosylation sites and their functional consequences are summarized in Table 6. These results underscore that site-specific S-nitrosylation acts as a critical regulatory switch, wherein S-nitrosylation promotes Src activity^[22]^, which in turn enhances EGFR phosphorylation and nuclear or mitochondrial translocation, thereby amplifying downstream transcriptional programs that drive cancer progression and therapeutic resistance^[41,42]^.

**Table 6:** The enzyme, modification sites, function of EGFR S-nitrosylation

**Modification types**	**Enzyme**	**Modification sites**	**Function**	**Ref.**
S-nitrosylation	NO donor	C166/C305	Inhibiting phosphorylation of the EGFR and its proliferative effects	[109]
	S-NO	C311	Promoting EGFR translocation to the plasma membrane and preventing the ER accumulation	[110]
	NOS2	/	Activating the EGFR and Src signaling pathway	[22]

EGFR: Epidermal growth factor receptor; NO: nitric oxide; NOS2: nitric oxide synthase 2; Src: SRC proto-oncogene, non-receptor tyrosine kinase.

### Targeting nitrosylation as a therapeutic strategy in TKI resistance

By stabilizing EGFR and enriching its active pool at the plasma membrane, S-nitrosylation likely contributes to the “membrane-retained” subtype of TKI resistance^[22]^. Therefore, inhibiting NO production represents a potential combinatorial strategy against TKI-resistant LC. For example, the NOS2 inhibitor aminoguanidine blocks NO-dependent EGFR nitrosylation, suppressing downstream signaling^[22]^. However, despite reaching Phase III trials (e.g., ACTION I/II), aminoguanidine development was discontinued due to safety concerns^[111,112]^. While the clinical development of aminoguanidine was halted, its mechanistic validation underscores the therapeutic potential of disrupting pathological S-nitrosylation^[22]^. Future efforts should focus on: (1) developing novel, more selective NOS2 inhibitors with improved safety profiles^[113]^; (2) exploring NO scavengers or targeted delivery systems (e.g., nanoparticles) to locally modulate tumor NO levels while minimizing systemic toxicity^[114]^; and (3) employing anti-inflammatory agents to suppress the tumor microenvironment-driven NOS2 upregulation that fuels this resistance pathway^[115]^. These approaches represent promising avenues to overcome this specific facet of TKI resistance. Key inhibitors targeting EGFR modifications discussed above are summarized in Table 7.

**Table 7:** The inhibitors targeting different PTMs and different spatial subtypes of EGFR

**Inhibitors**	**Targeting protein**	**Modification**	**Spatial subtypes**	**Application**	**Ref.**
Dasatinib	SFKs/c-Src	Phosphorylation	Nuclear and mitochondrial localization	Approved	[51]
T315	EGFR	Phosphorylation	Membrane-retained/ degradation-evading	Preclinical	[13]
Lutein	DHHC20	Palmitoylation	Membrane-retained	Nutritional Supplement	[59,62]
5-hydroxyflavone				Preclinical	
6-hydroxyflavone				Preclinical	
GKVL-TAT	ARF6	Palmitoylation	Membrane-retained	Preclinical	[18]
Orlistat	FASN	Palmitoylation	Membrane-retained/Nuclear and mitochondrial localization	Approved	[25,60]
9-ethyloxyimino9H-indeno[1,2-b]pyrazine-2,3-dicarbonitrile	USP8	Ubiquitination	Degradation-evading	Preclinical	[78,81]
NGI-1	STT3B	Glycosylation	Membrane-retained	Preclinical	[93]
Cisplatin	FUT8	Glycosylation	Membrane-retained	Approved	[87,97]
3Fax-Peracetyl Neu5Ac	ST6GAL1	Glycosylation	Membrane-retained	Preclinical	[94]
ACY-1215	HDAC6	Acetylation	Degradation-evading	Phase I/II	[104]
ACY-241			Degradation-evading	Phase I/II	
KA2507			Degradation-evading	Phase I/II	
JBI-802			Degradation-evading	Phase I/II	
FT-6876	CBP/p300	Acetylation	Degradation-evading	Preclinical	[106]
Aminoguanidine	NOS2	Nitrosylation	Membrane-retained	Phase III (Discontinued)+56	[22]

EGFR: Epidermal growth factor receptor; SFKs: SRC family kinases; c-Src: SRC proto-oncogene, non-receptor tyrosine kinase; DHHC20: DHHC-type palmitoyltransferase 20; ARF6: ADP-ribosylation factor 6; FASN: fatty acid synthase; USP: Ubiquitin-Specific Peptidase; FUT: fucosyltransferase; ST6GAL1: ST6 Beta-Galactoside Alpha-2,6-Sialyltransferase 1; HDAC6: histone deacetylase 6; CBP: CREB-binding protein; NOS2: nitric oxide synthase 2; p300: E1A binding protein p300; STT3B: STT3 oligosaccharyltransferase complex catalytic subunit B.

## THE PTM CROSSTALK NETWORK: A SYSTEMS VIEW OF EGFR FUNCTION AND THERAPEUTIC RESISTANCE

**Figure 8:**
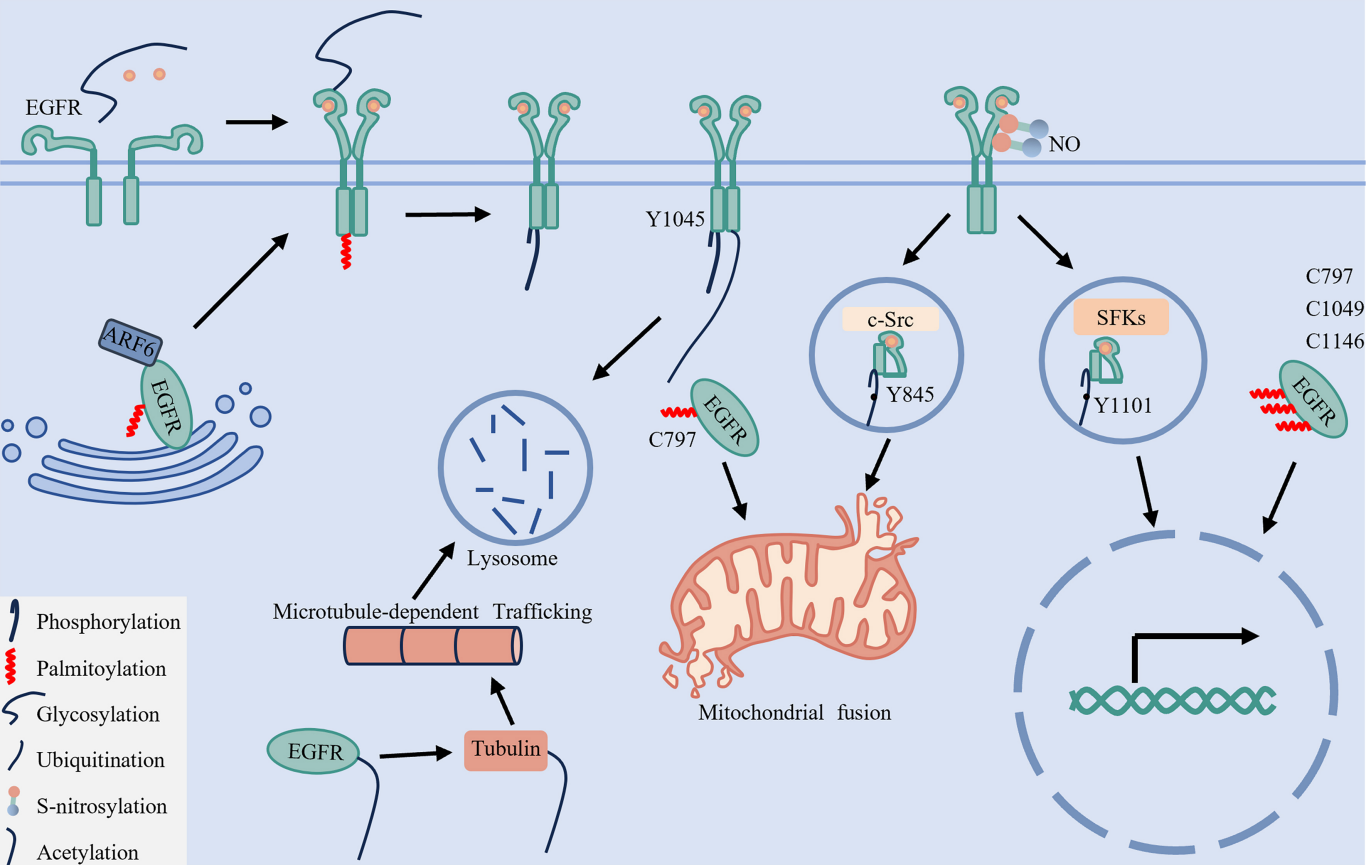
The PTM crosstalk regulatory network and spatial-fate model of EGFR. This diagram summarizes the cooperative regulation of EGFR subcellular localization and signaling through six key PTMs: phosphorylation, palmitoylation, glycosylation, ubiquitination, acetylation, and S-nitrosylation. ARF6-mediated palmitoylation facilitates EGFR trafficking from the Golgi apparatus to the plasma membrane. Glycosylation ensures proper receptor folding and dimerization, while together with palmitoylation, it supports stable membrane localization and enables downstream signaling. Phosphorylation at Y1045 serves as a molecular switch to initiate ubiquitination, whereas acetylation of EGFR promotes α-tubulin acetylation, which ultimately facilitates lysosomal degradation and signal termination. S-nitrosylation enhances Src binding to EGFR, promoting phosphorylation at Y845 and Y1101, thereby driving mitochondrial and nuclear translocation, respectively. Concurrently, palmitoylation at C797 together with phosphorylation at Y845 maintains mitochondrial localization, while phosphorylation at Y1101 combined with palmitoylation at C797, C1049, and C1146 sustains nuclear localization. EGFR: Epidermal growth factor receptor; ARF6: ADP-ribosylation factor 6; Src: SRC proto-oncogene, non-receptor tyrosine kinase; PTMs: post-translational modifications; SFKs: SRC family kinases; NO: nitric oxide.

While previous sections have detailed the regulation of EGFR by individual PTMs, these modifications do not function independently *in vivo*. Instead, they engage in a dynamic, interconnected crosstalk network that integrates diverse signals to precisely modulate receptor function, localization, and fate^[50]^. Thus, investigating the interplay within this PTM network - rather than examining each modification in isolation - is crucial for elucidating the mechanisms driving TKI resistance [Figure 8].

### The underlying order and interaction in PTM regulation

A fundamental feature of PTM crosstalk is its hierarchical and often reciprocal nature, in which one modification can serve as a molecular switch that triggers or modulates another.

Phosphorylation as a Primary Switch: Ligand-induced phosphorylation at Y1045 creates a docking site for the E3 ligase CBL, triggering receptor ubiquitination and subsequent lysosomal degradation^[48,67]^. Conversely, inhibition of this phosphorylation by TKIs disrupts degradation, contributing to the emergence of the “degradation-evading” resistance subtype^[13]^.

Pre-Kinase Modifications as Signal Triggers: Palmitoylation at Cys1025 anchors the C-terminal tail to the membrane, sterically hindering Grb2 binding and suppressing phosphorylation at Y1068, Y1148, and Y1173^[24]^. Glycosylation, required for proper receptor folding and membrane presentation, acts as an upstream permissive switch; its absence impairs ligand-induced autophosphorylation^[20]^. S-Nitrosylation at Cys166 and Cys305 enhances Src kinase activity, promoting EGFR phosphorylation at Tyr845 and Tyr1101 and thereby facilitating mitochondrial and nuclear translocation^[22,27,41,109]^.

### Cooperative coding: multimodal PTMs encode spatial fate

The ultimate destination and function of EGFR are determined by specific combinations of coordinated post-translational modifications, which collectively dictate its spatial fate.

Phosphorylation and Palmitoylation Direct EGFR Organelle Targeting: Mitochondrial targeting of EGFR requires both phosphorylation at Y845 and palmitoylation at Cys797^[25,46,60]^. Similarly, its nuclear translocation and function depend on phosphorylation at Y1101 together with palmitoylation at Cys797, Cys1049, and Cys1146^[25,41,61]^. These observations demonstrate that phosphorylation and palmitoylation function cooperatively as an integrated regulatory unit to guide EGFR to specific organelles.

Glycosylation and Palmitoylation Cooperate in EGFR Membrane Integrity: Proper plasma membrane localization and function of EGFR depend on synergistic contributions from glycosylation and palmitoylation. N-glycosylation ensures correct ectodomain folding and membrane presentation^[20,50,86]^, whereas palmitoylation is essential for efficient receptor trafficking from the Golgi to the plasma membrane through the ARF6-dependent pathway^[18]^.

Ubiquitination and Phophorylation in EGFR Signal Termination: EGFR degradation is initiated by phosphorylation at Y1045, which recruits CBL to mediate receptor ubiquitination. The resulting ubiquitin tag is then recognized by the ESCRT complex, directing sorted EGFR to lysosomes for degradation - the terminal step in signal termination^[48,67,79]^.

### Context-dependent network dynamics: metabolic and microenvironmental inputs

The PTM network is dynamic and continually reshaped by cellular context. External and internal cues reprogram this network through modulating substrate availability and enzyme activity.

Metabolic Coupling: Certain PTMs are directly coupled to cellular metabolism. For example, FASN-generated palmitate drives EGFR palmitoylation at mitochondria, a prerequisite for its subsequent phosphorylation and activation^[25,60]^. Similarly, flux through the hexosamine biosynthesis pathway - which supplies Uridine diphosphate N-acetylglucosamine (UDP-GlcNAc) for O-GlcNAcylation - can modify the ESCRT-0 component HGS, thereby impairing EGFR sorting to lysosomes^[92]^. Thus, oncogenic metabolic activity directly modulates the upstream PTM network.

Microenvironmental Regulation: The tumor microenvironment exerts selective pressure that reprograms the PTM network. For instance, hypoxia can upregulate sialyltransferases such as ST6GAL1, enhancing pro-survival sialylation of EGFR^[94,116]^. Inflammatory stress may elevate NOS2 expression, promoting S-nitrosylation and reinforcing Src/EGFR signaling^[22,109]^. Additionally, extracellular matrix stiffness can engage in crosstalk with EGFR signaling pathways. Collectively, the microenvironment acts as an upstream input layer that biases the network toward therapy-resistant states.

### Extending the PTM perspective: upstream regulation, multi-omic landscapes, and miRNA networks

The spatial and functional reprogramming of EGFR through PTMs is not an isolated event but is deeply embedded within a broader, multi-layered regulatory ecosystem. This ecosystem extends upstream to include the transcriptional and metabolic factors that control EGFR expression^[117-120]^, is reprogrammed at the genetic level by somatic alterations that reshape PTM networks^[27,121]^, and engages downstream in dynamic crosstalk with post-transcriptional circuits, particularly those involving microRNAs (miRNAs)^[122,123]^. A systems-level understanding of these interconnected layers is essential for developing comprehensive strategies to overcome TKI resistance.

Beyond directly modifying EGFR, PTMs also profoundly influence upstream regulatory factors that control EGFR expression, adding another layer of complexity to TKI resistance. For instance, Src acts as an upstream kinase that regulates JNK phosphorylation, which in turn phosphorylates c-Jun (Cellular Transcription factor AP-1 subunit Jun; at Ser63/73) within the Activator Protein 1 (AP-1) complex, enhancing its dimerization with c-Fos (Cellular Finkel-Biskis-Jinkins murine osteosarcoma virus oncogene homolog) and binding to EGFR gene enhancers to boost EGFR transcription^[117-120]^. Beyond transcriptional control, signal transducer and activator of transcription 3 (STAT3) activity is regulated by palmitoylation, which serves as a critical mechanism for its activation^[124]^. In the context of EGFR-TKI resistance, this enhanced activation of STAT3 could amplify the downstream STAT3-PARN [poly(A)-specific ribonuclease]-EGFR positive feedback loop, leading to sustained EGFR signaling and drug resistance^[125]^. Conversely, HDAC6-mediated deacetylation of p53 suppresses its transcriptional activity, thereby increasing EGFR protein stability^[126,127]^. Importantly, PTMs not only affect the expression and activity of EGFR but also regulate its upstream factors, underscoring the critical role of PTMs in mediating TKI resistance.

Integrating genomic data from next-generation sequencing (NGS) studies is crucial for understanding the genetic determinants of PTM network rewiring. Integrated analysis revealed that 5q deletion in basal-like breast cancer (identified by NGS) reduces SKP1 (S-phase kinase-associated protein 1) expression. SKP1 loss inactivates the SCF (Skp1-Cullin-F-box protein complex) ubiquitin ligase complex, leading to Src kinase stabilization and hyperactivity. Active Src, in turn, phosphorylates and activates EGFR, amplifying oncogenic signaling^[27,121]^. Integrated analyses from whole-exome sequencing in aggressive carcinomas suggest that co-occurring mutations in USP8 and EGFR may establish a synergistic “dual-hit” mechanism: gain-of-function USP8 mutations enhance deubiquitination, stabilizing EGFR protein by blocking its degradation, while concurrent EGFR kinase domain mutations drive constitutive kinase activity^[128]^. Furthermore, genetic sequencing reveals that mutant p53 exerts domain-specific gain-of-function effects: transactivation domain mutants promote cytoplasmic EGFR-AKT signaling, while DNA-binding domain mutants sustain nuclear EGFR activity by disrupting its phosphorylation of EGFR^[129]^. These findings underscore that somatic mutations can directly reprogram the EGFR signaling axis by affecting the enzymes and adaptors that constitute its PTM network.

Furthermore, the EGFR PTM network engages in reciprocal regulation with miRNA-mediated post-transcriptional circuits, forming a sophisticated layer of cross-talk. Under hypoxia, activated EGFR phosphorylates Argonaute 2 (AGO2) at Y393, disrupting the maturation of specific tumor-suppressive miRNAs (e.g., miR-31, miR-192) and thereby promoting cell survival^[122,123]^. Conversely, miRNAs can directly target components of the PTM machinery; for example, the EMT regulator miR-200f represses glycosyltransferases such as ST3GAL5 [ST3 beta-galactoside alpha-2,3-sialyltransferase 5; GM3 (monosialodihexosylganglioside) synthase], whose product GM3 modulates EGFR signaling - suggesting a feedback loop between EGFR glycosylation and miRNA expression^[130]^. Furthermore, DUBs such as USP8 play a key role in regulating the miRNA system by stabilizing AGO2. Concurrently, the ubiquitin system itself is subject to feedback regulation by miRNA networks^[73,123,131]^. This interconnectivity positions miRNAs both as effectors and modulators of the PTM network, revealing novel nodes for therapeutic intervention.

## DISCUSSION

The complexity and interplay of the multiple PTMs on EGFR are pivotal in triggering drug resistance during lung tumorigenesis. Here, we discuss how key PTMs - phosphorylation^[41]^, palmitoylation^[18]^, glycosylation^[20]^, ubiquitination^[78]^, acetylation^[21]^, and S-nitrosylation^[22]^ - collectively regulate EGFR signaling dynamics, protein stability, and subcellular localization. Rather than acting in isolation, these modifications cooperate to determine whether EGFR signals from the plasma membrane^[24]^, translocates to the nucleus or mitochondria^[25]^, or is targeted for degradation^[19]^. TKI therapy represents not merely kinase inhibition, but a profound perturbation of this homeostatic PTM network - suppressing certain axes while unmasking or amplifying others^[12]^. We therefore propose a classification framework based on the subcellular localization and stability of EGFR, categorizing resistance into four distinct subtypes: membrane-retained^[24]^, degradation-evading^[19]^, nuclear-localized, and mitochondrial-localized EGFR^[25]^.

Our spatial classification provides a valuable conceptual framework for understanding resistance heterogeneity. However, we acknowledge that in clinical specimens, these subtypes may not be mutually exclusive and can coexist within the same tumor. This complexity does not invalidate the model but rather underscores the need for high-resolution molecular profiling. Emerging technologies such as single-cell proteogenomics^[12]^ and multiplexed spatial imaging^[28]^ will be essential to delineate the distribution and co-occurrence of PTM-defined subpopulations *in situ*.

Therapeutically, the coexistence of subtypes suggests several strategic approaches: first, targeting the dominant subtype identified through biopsy analysis; second, inhibiting upstream regulatory nodes shared across multiple subtypes (e.g., SFK inhibitors such as dasatinib^[51]^ or FASN inhibitors such as orlistat^[25]^); and third, employing sequential or rational combination therapies guided by dynamic monitoring of resistance evolution via liquid biopsy.

We note that current clinical diagnostics remain limited in their ability to resolve these PTM-based subtypes routinely^[132]^. Future efforts should focus on translating PTM-spatial profiling into clinically applicable assays^[133]^. Furthermore, while our model emphasizes PTM-driven heterogeneity, tumor microenvironmental factors (e.g., hypoxia) also shape EGFR signaling and may interact with the PTM network - an area warranting integrated investigation^[116]^. Ultimately, moving beyond simple kinase inhibition to reprogram the pathological PTM network represents a promising paradigm for overcoming TKI resistance in LC.

## DECLARATIONS

### AI and AI-assisted tools statement

During the preparation of this manuscript, the AI tool DeepSeek was used solely for language editing. The tool did not influence the study design, data collection, analysis, interpretation, or the scientific content of the work. All authors take full responsibility for the accuracy, integrity, and final content of the manuscript.

### Copyright

© The Author(s) 2026.
